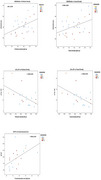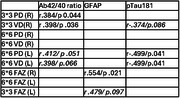# Association of Retinal Perfusion with Plasma Biomarkers of Alzheimer's Disease

**DOI:** 10.1002/alz70856_105606

**Published:** 2026-01-08

**Authors:** Sunu Mathew, Devin D Mackay, Jeffrey L. Dage, Fredrick Unverzagt, Kristen A. Russ, Kaj Blennow, Henrik Zetterberg, Elodie Foster, Kathy Howard, Aaron Vosmeier, Savannah Hottle, David G. Clark, Martin R. Farlow, Jared R. Brosch, Sujuan Gao, Liana G. Apostolova, Andrew J. Saykin, Shannon Risacher

**Affiliations:** ^1^ Indiana University School of Medicine, Indianapolis, IN, USA; ^2^ Department of Neurology, Indiana University School of Medicine, Indianapolis, IN, USA; ^3^ Department of Medical and Molecular Genetics, Indiana University School of Medicine, Indianapolis, IN, USA; ^4^ Stark Neurosciences Research Institute, Indiana University School of Medicine, Indiana, IN, USA; ^5^ Indiana Alzheimer's Disease Research Center, Indianapolis, IN, USA; ^6^ Indiana University, Indianapolis, IN, USA; ^7^ Indiana Alzheimer's Disease Research Center, Indiana University School of Medicine, Indianapolis, IN, USA; ^8^ University of Gothenburg, Mölndal, Sweden; ^9^ Institute of Neuroscienace and Physiology, University of Gothenburg, Mölndal, Västra Götaland, Sweden; ^10^ Institute of Neuroscience and Physiology, Sahlgrenska Academy at the University of Gothenburg, Gothenburg, Sweden; ^11^ Department of Radiology and Imaging Sciences, Indiana University School of Medicine, Indianapolis, IN, USA; ^12^ Regenstrief Institute, Inc, Indianapolis, IN, USA; ^13^ Center for Neuroimaging, Indiana University School of Medicine, Indianapolis, IN, USA; ^14^ Stark Neurosciences Research Institute, Indiana University School of Medicine, Indianapolis, IN, USA

## Abstract

**Background:**

The eye has been considered a ‘window to the brain’ and to several neurodegenerative brain disorders including Alzheimer's disease (AD) that display alterations in the eye, especially the retina. Plasma levels of AD biomarkers, including Aβ42/Aβ40 ratio, pTau 181, glial fibrillary acidic protein (GFAP), total Tau (tTau), and Neurofilament lightchain (NfL) are significantly altered in AD patients. We sought to evaluate the association of retinal perfusion measured using optical coherence tomography angiography (OCTA) with plasma biomarkers of AD.

**Method:**

Participants (31; 5 mild cognitive decline/AD, 6 subjective cognitive decline and 20 cognitively normal) underwent ophthalmological evaluation including OCTA and a blood sample. Single molecule array (Simoa) assays were used to measure plasma concentrations of Aβ42, and Aβ40, pTau181, GFAP, Ttau, and NfL. Partial Pearson correlations, covaried for age and sex, were used to compare retinal vessel and perfusion density with plasma level of the Aβ42/Aβ40 ratio, pTau181, GFAP, tTau and NfL.

**Result:**

Plasma Aβ42/Aβ40 showed a significant positive association with retinal vessel density (r=0.398 *p* = 0.036) and perfusion density (*r* = 0.384 *p* = 0.044). pTau 181 showed a significant negative association with retinal perfusion density (r=‐0.499 *p* = 0.041). GFAP showed a significant positive association with foveal avascular zone area in the superficial capillary plexus (r=0.554 *p* = 0.021).

**Conclusion:**

The majority of the sample was cognitively normal or mildly impaired, suggesting that retinal perfusion may be a useful tool for early diagnosis of AD‐related pathophysiology. Future longitudinal studies in larger samples and evaluating the utility of combining retinal and plasma biomarkers for predicting future progression to AD are needed.